# Involvement of *S*-adenosylmethionine-dependent halide/thiol methyltransferase (HTMT) in methyl halide emissions from agricultural plants: isolation and characterization of an HTMT-coding gene from *Raphanus sativus *(daikon radish)

**DOI:** 10.1186/1471-2229-9-116

**Published:** 2009-09-01

**Authors:** Nobuya Itoh, Hiroshi Toda, Michiko Matsuda, Takashi Negishi, Tomokazu Taniguchi, Noboru Ohsawa

**Affiliations:** 1 Department of Biotechnology, Faculty of Engineering (Biotechnology Research Center), Toyama Prefectural University, 5180 Kurokawa, Imizu, Toyama 939-0398, Japan

## Abstract

**Background:**

Biogenic emissions of methyl halides (CH_3_Cl, CH_3_Br and CH_3_I) are the major source of these compounds in the atmosphere; however, there are few reports about the halide profiles and strengths of these emissions. Halide ion methyltransferase (HMT) and halide/thiol methyltransferase (HTMT) enzymes concerning these emissions have been purified and characterized from several organisms including marine algae, fungi, and higher plants; however, the correlation between emission profiles of methyl halides and the enzymatic properties of HMT/HTMT, and their role in vivo remains unclear.

**Results:**

Thirty-five higher plant species were screened, and high CH_3_I emissions and HMT/HTMT activities were found in higher plants belonging to the Poaceae family, including wheat (*Triticum aestivum *L.) and paddy rice (*Oryza sativa *L.), as well as the Brassicaceae family, including daikon radish (*Raphanus sativus*). The in vivo emission of CH_3_I clearly correlated with HMT/HTMT activity. The emission of CH_3_I from the sprouting leaves of *R. sativus*, *T. aestivum *and *O. sativa *grown hydroponically increased with increasing concentrations of supplied iodide. A gene encoding an *S*-adenosylmethionine halide/thiol methyltransferase (HTMT) was cloned from *R. sativus *and expressed in *Escherichia coli *as a soluble protein. The recombinant *R. sativus *HTMT (RsHTMT) was revealed to possess high specificity for iodide (I^-^), bisulfide ([SH]^-^), and thiocyanate ([SCN]^-^) ions.

**Conclusion:**

The present findings suggest that HMT/HTMT activity is present in several families of higher plants including Poaceae and Brassicaceae, and is involved in the formation of methyl halides. Moreover, it was found that the emission of methyl iodide from plants was affected by the iodide concentration in the cultures. The recombinant RsHTMT demonstrated enzymatic properties similar to those of *Brassica oleracea *HTMT, especially in terms of its high specificity for iodide, bisulfide, and thiocyanate ions. A survey of biogenic emissions of methyl halides strongly suggests that the HTM/HTMT reaction is the key to understanding the biogenesis of methyl halides and methylated sulfur compounds in nature.

## Background

Methyl chloride (CH_3_Cl) exists in the atmosphere in large quantities (550 parts per trillion by volume, pptv) [1] due to its release from specific plants [2-4], fungi [5], and the burning of biomass [6]. Methyl bromide (CH_3_Br) was previously used as a soil fumigant, but its use is presently prohibited because it strongly depletes stratospheric ozone [7]. CH_3_Br (9 pptv in the atmosphere) [1] is also known to originate from oceanic sources [8], terrestrial plants [9,10], and the burning of biomass [6]. Thus, CH_3_Cl and CH_3_Br are the primary carriers of natural chloride and bromide to the stratosphere, where they catalyze ozone destruction. Compared with CH_3_Cl and CH_3_Br, which have long half-lives in the atmosphere of 1.0 and 0.7 years, respectively [1], methyl iodide (CH_3_I) has a much shorter half-life of 7-8 days [1,11]. However, methylene iodide (CH_2_I_2_) has recently been found to affect the formation of marine aerosols and cloud condensation nuclei [12], and iodine oxide (IO) causes ozone loss in the marine boundary layer [13]. CH_3_I (5-10 pptv in oceanic air), which is the most abundant biogenic methyl halide formed by the ocean [12,13], is expected to have the same effects as CH_2_I_2 _and is likely to be a carrier of iodide from the ocean to land. Although methyl halides (CH_3_X) are simple halogenated compounds that are mainly released from oceanic and terrestrial spheres as well as from anthropogenic sources, specific information about the origins, quantities generated, chemical and biosynthetic mechanisms, and physiological functions of methyl halides remains to be insufficient.

Wuosmaa and Hager [14] have reported that a chloride methyltransferase (*S*-adenosylmethionine: halide ion methyltransferase, HMT) from the marine red alga *Endocladia muricata *can transfer a methyl group from *S*-adenosyl-L-methionine (SAM) to a halide ion as follows:



This enzyme has also been found in a variety of organisms including higher plants [15-18], macro/micro algae [19,20], and soil bacteria [21]. In the same manner, *S*-adenosylmethionine: halide/thiol methyltransferase (HTMT) catalyzes the formation of methanethiol (CH_3_SH) and methyl thiocyanate (CH_3_SCN) in the presence of the bisulfide ion ([SH]^-^) or thiocyanate ion ([SCN]^-^) as follows [22-24]:





CH_3_Cl emissions have been reported from specific tropical plants, including certain types of fern, members of the family Dipterocarpaceae [2] and salt marsh plants [3]. On the other hand, CH_3_I emissions have been reported from marine algae, such as *E. muricata*, *Papenfusiella kuromo*, and *Sargassum horneri *(macroalgae) [14,19], *Pavlova *sp. (microalgae) [20], and various soil microorganisms [21]. Therefore, it can be speculated that different types of HMT/HTMT may be present in these organisms.

HMT and HTMT genes have been cloned from several higher plants including *Batis maritima *(*BmMCT*) [16,17], *Brassica oleracea *(*BoTMT1 *and *BoTMT2*) [25], and *Arabidopsis thaliana *(*AtHOL*) [18]. Their functions in these plants have been speculated to include salt-tolerance via the emission of methyl halides [15,16], and detoxification of sulfur compounds produced from the degradation of glucosinolates [24], although their precise roles in vivo remain unclear due to the lack of information available regarding these enzymes.

In this study, the extractable HMT/HTMT activity was measured in several agricultural plants as well as coastal trees and grasses. High HMT/MTMT activity was found in specific plants including *Raphanus sativus *(daikon radish), *O. sativa *(paddy rice), *T. aestivum *(wheat), and *Cyathea lepifera *(fern). Moreover, the gene encoding HTMT was isolated from *R. sativus *and expressed in *Escherichia coli*. This paper reports the emission profiles of methyl halides from some plants and the characterization of the enzymatic properties of recombinant *R. sativus *HTMT (RsHTMT).

## Results and discussion

### Distribution of HMT/HTMT activity in higher plants

To examine the distribution of HMT/HTMT activity in higher plants, HMT activity in crude extracts from 35 higher plants were assayed using the iodide ions (I^-^). Iodide is the most readily methylated ion among HMT/HTMT substrates. As shown in Table 1, the HMT activity was evaluated in several major agricultural plants, including *T. aestivum *(wheat), *O. sativa *(paddy rice), *Zea mays *(maize), and *Saccharum *sp. (sugar cane) from the Poaceae family, *R. sativus *and *Brassica napus *L. (rapeseed) from the Brassicaceae family, and *Basella alba *'Rubra' (*B. rubra*) from the Basellaceae family. Trace activities of less than 1 U (detection limit) were observed in a few coastal plants including *Arundo donax *L. (Poaceae), *Artemisia fukudo*, and *Suaeda maritima *(Table 1). Saini et al. [26] reported in their survey of methyl halides in higher plants that *B. napus *and *R. sativus *(Brassicaceae) have high in vivo HMT activity, *B. rubra *has medium activity, and *Z. mays *has low activity. The data obtained in the present study were similar to those reported by Saini et al., except that in the present study, *Glaux maritima *showed no methyl halide emissions. The present report is the first description of HMT activity in *T. aestivum *L. (wheat), which is a major crop species belonging to the Poaceae family. HMT/HTMT activity was observed in most members of the Poaceae and Brassicaceae families but only in a few species outside of these families. In addition, it was found that the CH_3_Cl-producing fern *Cyathea lepifera *[2] possessed HMT activity.

**Table 1:** HMT/HTMT activities in selected higher plants.

Plant	Activity (U/g fresh tissue)*
Agricultural plants	
Family Brassicaceae	
***Brassica campestris *(rapifera group**; leaf)**	1,700
***Brassica campestris *L**. (root)	1,400
***Brassica campestris *(pekinensis group**; leaf)	1,900
*Brassica napus L*. (sprouting leaf) JP26148	2,600
*Brassica oleracea *(italica group)	1,300
*Raphanus sativus *(mature leaf)	3,000
*Raphanus sativus *(mature root)	0
*Raphanus sativus *(sprouting leaf) JP26972	3,600
*Raphanus sativus *(sprouting stem) JP26972	400
*Raphanus sativus *(sprouting root) JP26972	82
	
Family Poaceae	
*Oryza sativa *(sprouting leaf) JP222429	120
***Triticum aestivum *L. Thell **(sprouting leaf) JP20300	210
***Saccharum sp*. L**. JP172543	~1
*Zea mays *L. (sprouting leaf) JP846	~1
	
Family Basellaceae	
*Basella rubra*	24

Seaside plants	
***Artemisia fukudo Makino***	~1
***Arundo donax L. var. gracilis Hack ***(Poaceae)	~1
*Suaeda maritima var. australia (R.Br.) Domin*	~1

Fern	
***Cyathea lepifera***	280

Agricultural plants including *O. sativa *L. (paddy rice), *Z. mays *L. (maize), ***T. aestivum *(L.) Thell **(common wheat), *B. napus *L. (rapeseed), and *R. sativus *L. (daikon radish) were cultured hydroponically from seeds. In the case of ***Saccharum *sp.**(sugar cane), cut stems were cultured in soil. Other plants examined in the survey of HMT/HTMT activity were collected from the Himi Seaside Botanical Garden (Himi, Toyama, Japan) or supplied by local farmers. HMT/HTMT activity was assayed with the crude extracts prepared from each plant tissue. No activity was observed in the following plants (the extracts were obtained from leaf samples, unless otherwise indicated): agricultural plants: ***Allium sativum *L**. (root), *Allium tuberosum*, ***Arctium lappa ***(root), ***Calysctegia soldanella***, ***Corchorus olitorius***, ***Cucumis sativus ***(fruit), ***Daucus carota ***(root), ***Dioscorea opposita ***(root), ***Elatostema umbellatum var. majus***, *Glycine max *L. Merr, *Impomea batatas *(root), ***Zingiber officinale ***(root), *Lactuca sativa*, *Solanum tuberosum *(root), seaside plants: ***Ascostichum aureum *L**., ***Bruguiera gymnorrhiza *L. Savigny**, ***Chrysanthemum crassum***, ***Chrysanthemum pacificum *Nakai**, ***Chrysanthemum shiogiku****, Glaux maritima var. obtusifolis *Fern., ***Kandelia candel *L. Druce**, ***Rhizophora stylosa *Griffith**, ***Rubus trifidus *Thunb**, ***Triglochin maritimum***, ***Vitex rotundifolia *Linn. fil.**, ***Xylocarpus granatum *(Lin.) Koenig**.

* The mean value of duplicate samples.

**The plants whose HMT/HTMT activity was first analyzed in this work are written in bold letters.

Leaves of young *R. sativus *seedlings (3-5 days old) exhibited the highest HTMT activity (ca. 3,600 U/g fresh leaves) among the plants tested. The enzymatic properties of the HTMT enzyme in *R. sativus *have not yet been investigated; therefore, *R. sativus *leaves were used for further enzymatic experiments. The HTMT activity of *R. sativus *was primarily localized in the leaves, with activity in the stem and young roots much weaker, and no activity detected in the mature *R. sativus *root. In contrast, a similar level of HTMT activity was detected in *B. campestris *L. (rapifera group) roots compared with leaves. Attieh et al. [25] have reported that stronger thiol methyltransferase activity was observed in leaves than stems and roots in young seedlings and much weaker activity was found in mature plants in cabbage (*B. oleracea*). On the other hand, *AtHOL1 *of *A. thaliana*, which is a homologous gene of *BoTMT1 *in *B. oleracea*, is ubiquitously expressed during growth and *AtHOL3 *is highly expressed in roots of mature plants [27]. These findings suggest that *R. sativus *has a unique activity profile of HTMT compared with other Brassicaceae plants.

### Emission profiles and rates of CH_3_I from *T. aestivum*, *O. sativa *and *R. sativus*

**Figure 1:**
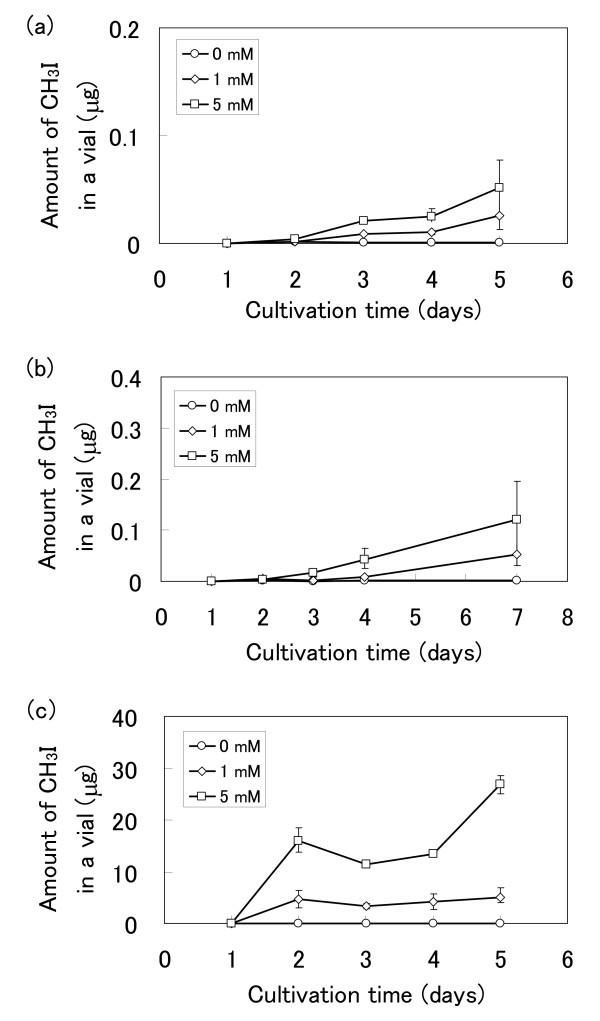
**Emission profiles of methyl iodide from (a) *T. aestivum*, (b) *O. sativa *and (c) *R. sativus *grown in hydroponic culture with 0--5 mM potassium iodide**. Values are shown as the mean ± standard deviation of three replicate samples.

**Figure 2:**
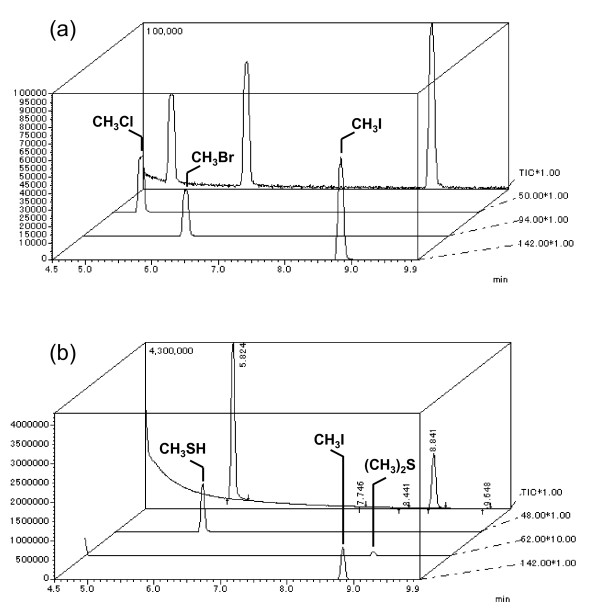
**GC-MS analysis of methyl halides, methanethiol, and DMS from *R. sativus***. (a) GC-MS spectrum of methyl halide standards (5 ppm each), total ion chromatogram (TIC) of methyl halides (background), and selected ion chromatogram of each methyl halide (foreground). (b) Emission products from *R. sativus *cultured with 5 mM potassium iodide for 4 days.

The emission profiles of CH_3_I from three plant species were measured (Figure 1). These plants were cultured hydroponically to avoid the effect of soil microorganisms. No CH_3_I emissions were detected in the absence of I^-^; however, the emission levels rose in response to increasing concentrations of iodide ions ranging from 1 mM to 5 mM. This indicates that free I^- ^in water are crucial for the formation of CH_3_I, and that CH_3_I emission is affected by iodide concentration. In vivo production of CH_3_I was observed from *T. aestivum*, *O. sativa *and *R. sativus *when I^- ^were supplied and the formation of CH_3_SH and dimethyl sulfide (DMS) was always detected in GC-MS analyses (Figure 2) in the absence of halide ions. This data concurs with the previous reports by Fall et al. [28] and Kanda et al. [29,30] of the emission of sulfur-containing gases including DMS from maize, wheat, and rice.

The worldwide areas of rice and wheat cultivation are approximately 140-150 × 10^6 ^and 210-220 × 10^6 ^ha, respectively. It is therefore important to evaluate the level of emissions of volatile compounds, such as methyl halides, from these plants and to clarify the mechanism of synthesis of these compounds. The emission rates of CH_3_I from these plants in the presence of 5 mM iodide were 0.4 (*T. aestivum*), 3.1 (*O. sativa*), and 30.8 μg/g fresh leaf per day (*R. sativus*), and correlated with the observed HMT/HTMT activities (*T. aestivum*, 210; *O. sativa*, 120; *R. sativus*, 3,600 U/g fresh leaf). The results of this study confirm that methyl halide emissions from rice and wheat plants are dependent on HMT/HTMT activity. The slight differences between the emission rates of *T. aestivum*, *O. sativa*, and *R. sativus *and their HMT/HTMT activities are probably due to the specific properties of the HMT/HTMT in these plants, especially their *K*_m _values towards I^-^. Saini et al. [26] have reported that CH_3_I emission from leaf disks of *B. oleracea *was 168.3 μg/g fresh leaf per day in the presence of 50 mM iodide. This value is comparable with that obtained for *R. sativus *in this study, although the experimental conditions between the studies differed.

In vivo emission of CH_3_Cl or CH_3_Br from *R. sativus *was observed when plants were supplemented with Cl^- ^or Br^-^, and CH_3_Cl or CH_3_Br was detected in the in vitro reactions using the crude enzyme preparation (Table 2). However, no emissions of CH_3_Cl or CH_3_Br from *T. aestivum *and *O. sativa *were observed in vivo or in vitro due to the low levels of HMT activity in these plants. Muramatsu and Yoshida [31] first confirmed the emission of CH_3_I from rice paddies, and Redeker et al. [32] also detected emissions of methyl halides, mainly CH_3_I, from the same ecosystem involving soil, soil microorganisms, and rice plants. More recently, Redeker et al. [33] analyzed the methyl halide emissions from rice plants in more detail. A hydroponic system was adopted in the present study so that emissions reflected those of the tested plants alone, and were not affected by the presence of soil microorganisms. The results of the present study, together with the report of an HMT homologue in rice [18], indicate that rice plants produce CH_3_I through an HMT/HTMT reaction, and soil microorganisms mainly play a role in liberating I^- ^from the soil, where it is present as iodate (IO_3 _^-^). The mean concentration of iodide in field soil ranges from 5 to 20 mg/kg in dry soil, and around 2 mg/kg dry soil in paddy soil [31]. This difference is explained by an increase in the reductive conditions of the paddy when flooded. Stable forms of iodate (IO_3 _^-^) adsorbed onto the soil matrix may be reduced by microorganisms in flooded paddies to give soluble I^-^, which could then be taken up through roots and used as an HMT/HTMT substrate to form CH_3_I.

**Table 2:** Substrate specificity of HTMT from *R. sativus*.

Anion (mM)	Production rate of methyl compounds (pmol/min/mg protein)	
	
	*Raphanus sativus*	*Brassica oleracea*
Cl--(20)	N.D. *	--
(50)	6	--
Br--(20)	79	--
(50)	234	--
I--(20)	3,094	2,685
(50)	5,552	--
[SH]^- ^((NH_4_)_2_S) (20)	339	267
[SH]^- ^(NaSH) (20)	6,428	4,456
[SCN]^- ^(20)	1,028 **	645 **
[CN]^- ^(20)	N.D. *	N.D. *

*N.D., not detected.

** Measured from the amount of CH_3_SH converted from CH_3_SCN in the gaseous phase; the amount of CH_3_SCN in the liquid phase was negligible.

### Partial purification and anion-specificity of HTMT from *R. sativus*

**Figure 3:**
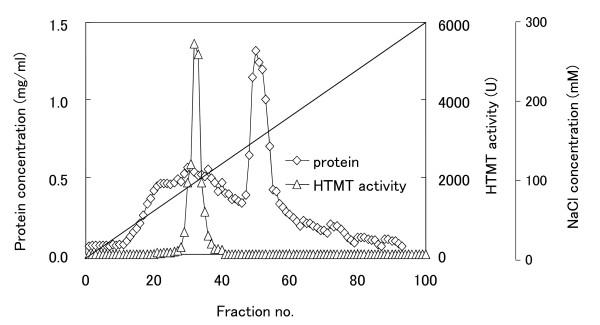
**DEAE anion exchange chromatography of HTMT from *R. sativus***. Triangles represent HTMT activity and diamonds represent protein concentration.

*B. oleracea *possesses several isoforms of thiol methyltransferases, which are able to catalyse the SAM-dependent methylation of iodide [23]. Therefore, the existence of HTMT isoforms in *R. sativus *was examined using partial enzyme purification by chromatography. As shown in Figure 3, one major bell-shaped HTMT activity peak was observed on the chromatogram, and most of the activity in the crude extract was recovered in this peak. This result indicates that the sprouting leaves of *R. sativus *produce one major HTMT isoform. Using the crude enzyme preparation of HTMT, the substrate specificity towards anions was measured, and compared with that of *B. oleracea*. As shown in Table 2, the enzyme from *R. sativus *exhibited the highest activity towards [SH]^- ^and I^-^, whereas the activities toward Br^- ^and Cl^- ^were much lower. The substrate spectrum of HTMT was consistent with the in vivo emission rates of methyl halides.

Attieh et al. [15,23] reported that HTMT from *B. oleracea*, which belongs to the same family as *R. sativus *(Brassicaceae), is able to methylate I^- ^as well as (NH_4_)_2_S ([SH]^-^) and [SCN]^-^. Because (NH_4_)_2_S and NaSH ([SH]^-^) react chemically with SAM to produce small amounts of CH_3_SH and/or DMS, the enzymatic formation of these products was analyzed carefully. It was confirmed that the HTMTs of *R. sativus *and *B. oleracea *possessed sulfide methylating activity towards NaSH. However, this activity was weak towards (NH_4_)_2_S (Table 2). This discrepancy between the present and previous data [15,23] could be due to differences in the experimental conditions. (NH_4_)_2_S is not a good substrate to measure the formation of CH_3_SH by HTMTs. In order to measure CH_3_SCN or CH_3_CN production by HTMT with KSCN or KCN as substrates, the reaction mixture was measured directly by GC-14A gas chromatography because most of the CH_3_SCN or CH_3_CN was dissolved in water and did not transfer into the gaseous phase. It was found that most of the CH_3_SCN produced by the HTMT reaction in *R. sativus *was converted to CH_3_SH by an unknown chemical reaction catalyzed by a protein (Table 2). In addition, it was confirmed that *R. sativus *exhibited methionine γ-lyase activity that produces CH_3_SH from methionine. These results indicate that CH_3_SH is possibly produced through several pathways in *R. sativus*. However, DMS production could not be detected with the HTMT reaction from SH^- ^and SAM. Rhew et al. [18] have reported that the emission of methyl halides by *A. thaliana *is inhibited by the addition of [SCN]^-^. The authors speculated that methyl halide emissions were competitively inhibited by [SCN]^- ^because this ion is the preferred substrate for HTMT in *A. thaliana*.

### Cloning and sequence analysis of an HTMT coding gene from *R. sativus*

To investigate the properties of HTMT in *R. sativus*, a full length HTMT-encoding gene (*Rshtmt*) was isolated. Degenerate PCR was performed to isolate a partial sequence of *Rshtmt *using total RNA prepared from sprouting leaves of *R. sativus *as a template. The PCR product gave a single fragment of 300 bp in size. The fragment was cloned into a pTA2 vector and the nucleotide sequence of the insert was determined. The amino acid sequence deduced from the nucleotide sequence indicated high similarity to HMT/HTMT genes of higher plants. In order to isolate the full length *Rshtmt *sequence, 3'/5'-RACE was performed using several primers designed with reference to this nucleotide sequence, and a single open reading frame (ORF) that encoded a HTMT was detected in the analyzed nucleotide sequence. The full length nucleotide sequences of the cDNA and genomic DNA containing *Rshtmt *were obtained by PCR amplification using specific primers.

A comparison of the cDNA and genomic sequences revealed that the *Rshtmt *ORF contains 7 introns. *Rshtmt *encodes a protein of 226 amino acid residues, and the deduced amino acid sequence showed a significant similarity to those of higher plant HMTs/HTMTs, which belong to family 11 of the methyltransferase superfamily, including *B. oleracea *BoTMT1 (GenBank: AF387791, 94.2% identity), *A. thaliana *AtHOL1 (GenBank: NP181919, 80.2%), *B. maritima *BmMCT (GenBank: AF109128, 64.8%), and *Z. mays *SAM-dependent methyltransferase (GenBank: EU956554, 54.8%]. The deduced amino acid sequence of *Rshtmt *contains several motifs and a secondary structure that is conserved among the *S*-adenosyl-L-methionine-dependent methyltransferases [34-37]. The results indicate that the cloned *Rshtmt *belongs to the *S*-adenosyl-L-methionine-dependent methyltransferase (SAM-MT) family. The prototypical SAM-MT fold is constructed of seven β strands (β1-β7) and six α helices (αZ and αA-αE) [37], although the β7 strand of RsHTMT is replaced by an α helix. Such structural differences might contribute to the substrate specificity of RsHTMT.

### Enzymatic properties of recombinant RsHTMT

**Figure 4:**
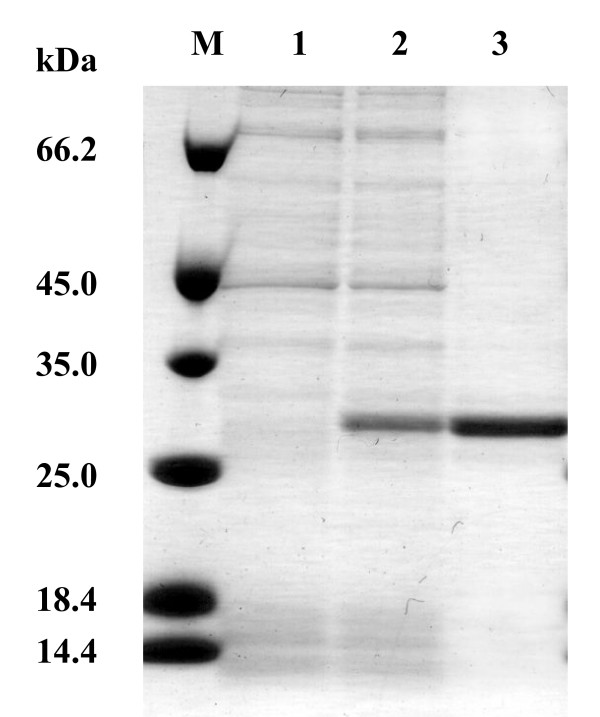
**SDS-PAGE analysis of recombinant RsHTMT**. Proteins were separated by SDS-PAGE and stained using Coomassie brilliant blue. M, Molecular marker; Lane 1, crude cell free extract of *E. coli *BL21(DE3); Lane 2, crude cell free extract of *E. coli *transformant possessing pET-Rshtmt; Lane 3, recombinant RsHTMT purified by Ni-Sepharose resin column chromatography.

To obtain recombinant RsHTMT, *Rshtmt *was introduced into *E. coli *BL21 (DE3) using the expression vector pET-21b. The recombinant RsHTMT was expressed as a histidine-(His-) tagged soluble protein in *E. coli *cells and purified by Ni-Sepharose resin column chromatography. The purified RsHTMT appeared homogenous, as judged by SDS-PAGE, and its molecular mass was estimated to be 29 kDa (Figure 4). This value is close to the molecular mass of 27.5 kDa predicted from the amino acid sequence of *Rshtmt *including the His-tag. The purified protein was characterized and its substrate specificity was determined. The *K*_*m *_values of recombinant RsHTMT for Cl^-^, Br^-^, I^-^, [SH]^-^, [SCN]^- ^and SAM were 1656.40 mM, 177.34 mM, 4.47 mM, 12.24 mM, 0.04 mM, and 0.19 mM, respectively, as shown in Table 3. The enzyme showed no activity towards CN^-^. Saini et al. [28] reported *K*_*m *_values for Cl^-^, Br^-^, I^-^, [SH]^-^, and SAM of *B. oleracea *thiol methyltransferase of 85 mM, 29 mM, 1.3 mM, 4.7 mM, and 0.03 mM, respectively. The values obtained for RsHTMT were therefore similar to *B. oleracea *thiol methyltransferase in terms of methyl acceptor preference: high specificity for I^-^, [SH]^-^, and [SCN]^-^, and low specificity for Cl^- ^and Br^-^. Purified RsHTMT showed a high specificity for [SCN]^-^, although much lower activity was found when a crude extract was used to assay the enzyme activity (Table 2). This discrepancy could be due to the existence of other proteins in the crude extract; however, the precise reason remains unclear. It is known that many enzymes are inhibited in the presence of high concentration of anions, such as bisulfide, thiocyanide, and halide ions [38-41]. Attieh et al. [25] reported that the expression pattern of thiol methyltransferases of *B. oleracea *corresponds to the concentration of glucosinolate. This suggests that RsHTMT in *R. sativus *may be involved in the detoxification of sulfur compounds produced by the degradation of glucosinolates to release them as volatile compounds. The volatile sulfur compounds, including CH_3_SH and CH_3_SCN and methyl halides, are believed to act as insecticidal or anti-pathogenic agents. Therefore, it is speculated that RsHTMT in *R. sativus *plays a role in controlling the levels of anions that can inhibit metabolic enzymes in the leaves and also to protect them from damage caused by insects or pathogens.

**Table 3:** Kinetic parameters of purified recombinant RsHTMT.

Substrate	*K*_m _(mM)	*V*_max _(pmol/min/mg)	*V*_max_/*K*_m_
SAM	0.19	--	--
Cl^-^	1657.40	3,381	2.04
Br^-^	177.34	34,965	1.97 × 10^2^
I^-^	4.47	139,286	3.12 × 10^4^
[SH]^- ^(NaSH)	12.24	158,732	1.30 × 10^4^
[SCN]^-^	0.04	185,185	4.41 × 10^6^

Kinetic parameters for SAM were measured at a constant iodide concentration (20 mM). Parameters for each of the methyl acceptors were measured at constant SAM concentration (500 μM).

## Conclusion

It was found that there is high HMT/HTMT activity in the sprouting leaves of *R. sativus *(daikon radish), *T. aestivum *(wheat), and *O. sativa *(paddy rice). HMT/HTMT activity was responsible for in vivo CH_3_I emissions from these agricultural plants. The *Rshtmt *gene was cloned successfully and expressed in *E. coli *cells. The activity data from purified RsHTMT suggest that RsHTMT may participate in sulfur metabolism in sprouting leaves of *R. sativus*. The HMT/HTMT reaction was found to be involved in the emission of methyl halides or volatile sulfur compounds from higher plants and is key to our understanding of the biogenesis of these compounds in nature.

## Methods

### Culture and collection of plants

Agricultural plants including *O. sativa *L. (paddy rice), *Z. mays *L. (maize), *T. aestivum *(L.) Thell (common wheat), *B. napus *L. (rapeseed), and *R. sativus *L. (daikon radish) were cultured hydroponically. Seeds were placed on cotton matrices supplemented with modified Hoagland's solution: 1 mM KH_2_PO_4_, 5 mM KNO_3_, 5 mM Ca(NO_3_)_2_.4H_2_O, 0.3 μM CuSO_4_.5H_2_O, 2 mM MgSO_4_.7H_2_O, 46 μM H_3_BO_3_, 24 μM Ferric-NaEDTA, 9 μM MnSO_4_.H_2_O, 0.1 μM NH_4_MoO_4_.4H_2_O, 0.7 μM ZnSO_4_.7H_2_O (pH 5.7). In the case of *Saccharum *sp. (sugar cane), cut stems were disinfected and placed in a pot with soil and cultured. Plants were grown at 20°C and 40 μE/m^2^/s (12 h light; 12 h dark) for *R. sativus*, *T. asetivum *and *B. napus*, and at 30°C and 133 μE/m^2^/s for *O. sativa*, *Z. mays *and *Saccharum *sp. between 4 and 15 days until enough leaves or blades were obtained.

Plant seeds (JP strains; Table 1) including rice, wheat, daikon radish and rapeseed and stems of sugar cane were supplied by the National Institute of Agrobiological Sciences (NIAS), Tsukuba, Japan. Other plants examined in the survey of HMT/HTMT activity were collected from the Himi Seaside Botanical Garden (Himi, Toyama, Japan) or supplied by local farmers.

### Crude enzyme preparations from plants

Plant tissue (1-2 g wet weight) containing mainly leaves was ground using sea sand (40-80 mesh) in a mortar and pestle at 4°C, and then extracted with 20 mM MES buffer (pH 7.0) containing 5 mM dithiothreitol (DTT) at a ratio of 0.1 g sample/0.5 ml buffer. The crude extract was centrifuged at 4°C for 30 min at 10,000 × *g *to obtain the supernatant. In the case of *R. sativus*, the supernatant was filtered prior to measuring CH_3_SH and DMS levels using an Econo-Pac 10DG gel filtration column (Bio-Rad) to eliminate endogenous CH_3_SH and DMS.

### Measurement of HMT/HTMT activity

The formation of methyl halides, CH_3_SH, and DMS was assayed using a Shimadzu QP-2010 gas chromatographer-mass spectrometer (GC-MS; quadrupole) equipped with a TurboMatrix HS40 head space sampler (Perkin Elmer). The enzyme solution was incubated in a 5 ml mixture containing 0.5 mM SAM, 20 or 50 mM halides (KX), or 20 mM (NH_4_)_2_S (pH 7.0); or NaSH for bisulfide methylation, and 20 mM MES (pH 7.0). Enzyme reactions were started by the addition of 0.2-1.0 ml of enzyme solution. The mixture was incubated in a 22-ml vial sealed using a silicon septum, followed by shaking at 170 rpm at 30°C for 30 min. The reaction was stopped by heating at 70°C for 5 min in a water bath. Each sample vial was then connected to the head space sampler and automatically held at 70°C for 20 min to transfer volatile compounds into the gaseous phase. The gas phase was drawn for 0.2 min after pressuring the tube for 3 min at 70°C to carry the sample gas into the GC-MS inlet. The temperature of the transfer line and syringe was maintained at 90°C. The head space gas was injected into a DB-VRX capillary column (J & W Scientific; 60 m × 0.25 mm i.d., 1.4 μm film thickness) for GC-MS analysis. The carrier gas (He) flow rate was 3.9 ml/min (100 kPa), and the linear velocity of the capillary column was 23.6 cm/s. Samples were injected automatically in splitless mode for 1 min at 180°C with the following column temperature program: 40°C for 5 min, 2°C/min increases to 50°C, and then 10°C/min increases to 180°C. Mass spectra were obtained at 70 eV using an electron-impact ion source (EI, 200°C). The retention times of CH_3_Cl, CH_3_Br, CH_3_I, CH_3_SH, and DMS were 5.05, 6.20, 9.00, 5.85, and 8.90 min, respectively. The products were quantified by peak area and identified by comparison with the retention times and molecular ions (m/z) of methyl halide, CH_3_SH, and DMS standards.

The formation of CH_3_SCN and CH_3_CN in the reaction mixture was measured by GC-14A gas chromatography (Shimadzu) using a flame ionization detector. The enzyme solution (50 μl) was added to a solution containing 0.5 mM SAM, 20 mM KSCN or KCN, and 20 mM MES (pH 7.0) in a total volume of 1 ml. After incubation at 170 rpm at 30°C for 30 min, the reaction was stopped by heating at 70°C for 5 min. A 5 μl aliquot of the reaction mixture was injected directly into a packed column (2.1 m × 3.2 mm) of Thermon1000/ShimaliteW (Shimadzu GLC Inc.; column T, 80°C; injection T, 140°C; detection T, 150°C; flow rate, 40 ml/min of N_2_) by GC. The retention times of CH_3_CN and CH_3_SCN were 1.48 and 4.48 min, respectively, and the products were quantified using the peak area.

To calibrate the concentrations of the products, gas and liquid standards of CH_3_I, DMS, and CH_3_SCN were prepared. The detection limits of the GC-MS analysis for methyl halides, CH_3_SH, and DMS were around 0.03 ppm in the gaseous phase, and that of the GC-14A for CH_3_SCN was 0.05 mM (3.66 ppm) in the liquid phase. The total amount of each product, except for CH_3_SCN and CH_3_CN, was calculated from the concentration of the gas phase, assuming that the equilibrium of each compound in air and water in a vial was attained. One unit (U) of enzyme activity was defined as the amount of the enzyme that catalyzed the formation of 1 pmol of methyl halides, CH_3_SH, or CH_3_SCN in one min at 30°C.

### Partial purification of HTMT from the sprouting leaves of *R. sativus*

The following procedures for purifying HTMT were all carried out at 4°C unless stated otherwise. The sprouting leaves of *R. sativus *were collected (10 g wet weight), ground using a mortar and pestle with sea sand (40-80 mesh), and extracted with 10 ml of Tris-HCl buffer (pH 7.5) supplemented with 5 mM DTT. In order to remove phenolic compounds, 10% (w/v) polyvinyl polypyrrolidone was added to the recovered supernatant. After centrifugation at 10,000 × *g *for 30 min, the supernatant was dialyzed with Tris-HCl buffer containing 5 mM DTT. The enzyme solution was applied to a DEAE-Toyopearl 650 M anion exchange column (28 × 45 mm, Tosoh Corp., Tokyo, Japan), which had been equilibrated with the above buffer. The enzyme was eluted with a 0-0.3 M NaCl linear gradient in buffer (total 400 ml). The HTMT activity was measured for all fractions obtained. The protein concentration was estimated by measuring the absorbance at 280 nm or using a Bio-Rad Protein Assay kit (Sigma Aldrich) with bovine serum albumin (BSA) as the standard protein in accordance with the manufacturer's protocol.

### Strains and vectors for genetic manipulation

*R. sativus *was used as a source of chromosomal DNA and total RNA for the isolation of the *Rshtmt *gene. *E. coli *JM109 cells and plasmid vector pTA2 were used in DNA manipulation. *E. coli *BL21(DE3) cells and expression vector pET-21b were used to express the recombinant RsHTMT in *E. coli*.

### Cloning of the HTMT coding gene from *R. sativus*

Standard techniques were used for DNA manipulation [42]. Genomic DNA and total RNA were isolated from the sprouting leaves of *R. sativus *grown on Hoagland's solution for 4 days. Genomic DNA was prepared by the method of Dellaporta et al. [43]. Total RNA was isolated using an RNeasy Plant Mini kit (Qiagen) according to the manufacturer's protocol. First strand cDNA was synthesized using a PrimeScript High Fidelity RT-PCR kit (TaKaRa) with an oligo dT primer, and the products were used as PCR templates. A set of degenerate oligonucleotide primers (sense primer, 5'-CTKGTMCCCGGMTGTGGY-3'; antisense primer, 5'-SAGRGTKATGAGYTCKCCRTC-3') were designed on the basis of partial amino acid sequences conserved among the higher plant thiol methyltransferase- and HMT-coding genes. In order to obtain the nucleotide sequences of the 3'- and 5'-ends of the HMT-coding cDNA, 3'- and 5'- rapid amplification of cDNA ends (RACE) was carried out using 3'/5'-Full RACE Core Set (TaKaRa) with first strand cDNA as a template. The whole genomic and cDNA fragments of *Rshtmt *were amplified by PCR using primers designed from the nucleotide sequence of the N- and C-termini. The nucleotide sequence of *Rshtmt *was determined using a capillary DNA sequencer 310 (Applied Biosystems) and was deposited in the DNA Data Bank of Japan (DDBJ) database under accession no. AB477013.

### Expression and purification of recombinant RsHTMT

The *Rshtmt *cDNA corresponding to the mature HTMT sequence was amplified by PCR using two oligonucleotide primers (sense primer, 5'-CCAT*GGATCC*AATGGCTGAGGGACAACA-3', *Bam*HI site in italics; antisense primer, 5'-GTCGACTTA*AAGCTT*GTTGATCTTTTTCCACCTACC-3', *Hin*dIII site in italics). The amplified fragment was digested with *Bam*HI and *Hin*dIII and ligated into the expression vector pET-21b treated with the same restriction enzymes. The resulting plasmid encoding a His-tagged translational fusion of RhHTMT was named pET-Rshtmt and was introduced into *E. coli *BL21 (DE3). Transformants were grown on LB medium containing 50 μg/ml ampicillin to OD_600 _0.4 at 37°C with shaking. Isopropyl-β-D-thiogalactoside (IPTG) was added to a final concentration of 1 mM to induce expression of the recombinant protein, and cells were incubated for a further 4 hours. The cells were harvested by centrifugation and resuspended in cell lysis buffer (20 mM MES, 0.5 M NaCl, 5 mM DTT, and 10 mM imidazole, pH 7.0). The cell suspension was sonicated five times for 30 s each and centrifuged at 15,000 rpm for 5 min. The supernatant was loaded onto a Ni-Sepharose™ high performance column (1 ml bed volume). The column was washed with 10 ml of cell lysis buffer and recombinant RsHTMT fused to the His-tag was eluted using elution buffer (20 mM MES, 0.5 M NaCl, 5 mM DTT, and 0.5 M imidazole, pH 7.0). Eluted fractions containing recombinant RsHTMT were desalted using an Econo-pac column with 20 mM MES buffer (pH 7.0) containing 5 mM DTT. The solution obtained was analyzed to determine the protein concentration and retained for further experiments.

### Chemicals

*S*-adenosyl-L-methionine (SAM) was obtained from Sigma. Gases containing CH_3_Cl, CH_3_Br, and CH_3_I (1 or 5 ppm in N_2_) were specially prepared by Sumitomo Seika Co., Osaka, Japan, and gases containing CH_3_SH and DMS (1 and 5 ppm in N_2_) were obtained from Takachiho Chemical Industrial Co., Tokyo.

## Abbreviations

HMT: *S*-adenosyl-L-methionine; halide ion methyltransferase; HTMT: *S*-adenosyl-L-methionine: halide/thiol methyltransferase; SAM: *S*-adenosyl-L-methionine; SAH: *S*-adenosyl-L-homocysteine; DMS: dimethyl sulphide.

## Authors' contributions

MM, TT, and NO carried out analysis of the emission profile of methyl halides from higher plants and partial purification of native HTMT from *R. sativus*. TN cloned the partial cDNA fragment that encoded HTMT from *R. sativus*. HT performed the isolation and heterologous expression of the HTMT encoding gene from *R. sativus *and characterization of the enzymatic properties of recombinant HTMT and wrote those sections. NI planned the experimental design and wrote the section on emission of methyl halides from plants.
